# Supplementary material for: Whole genome and transcriptome integrated analyses guide clinical care of pediatric poor prognosis cancers
Source: Nat Commun. 2024 May 16;15:4165. doi: 10.1038/s41467-024-48363-5 (PMC11099106; doi:10.1038/s41467-024-48363-5)
Supplement: Supplementary file 1 — Supplementary Information [file 41467_2024_48363_MOESM1_ESM.pdf]

Supplementary Figure 1

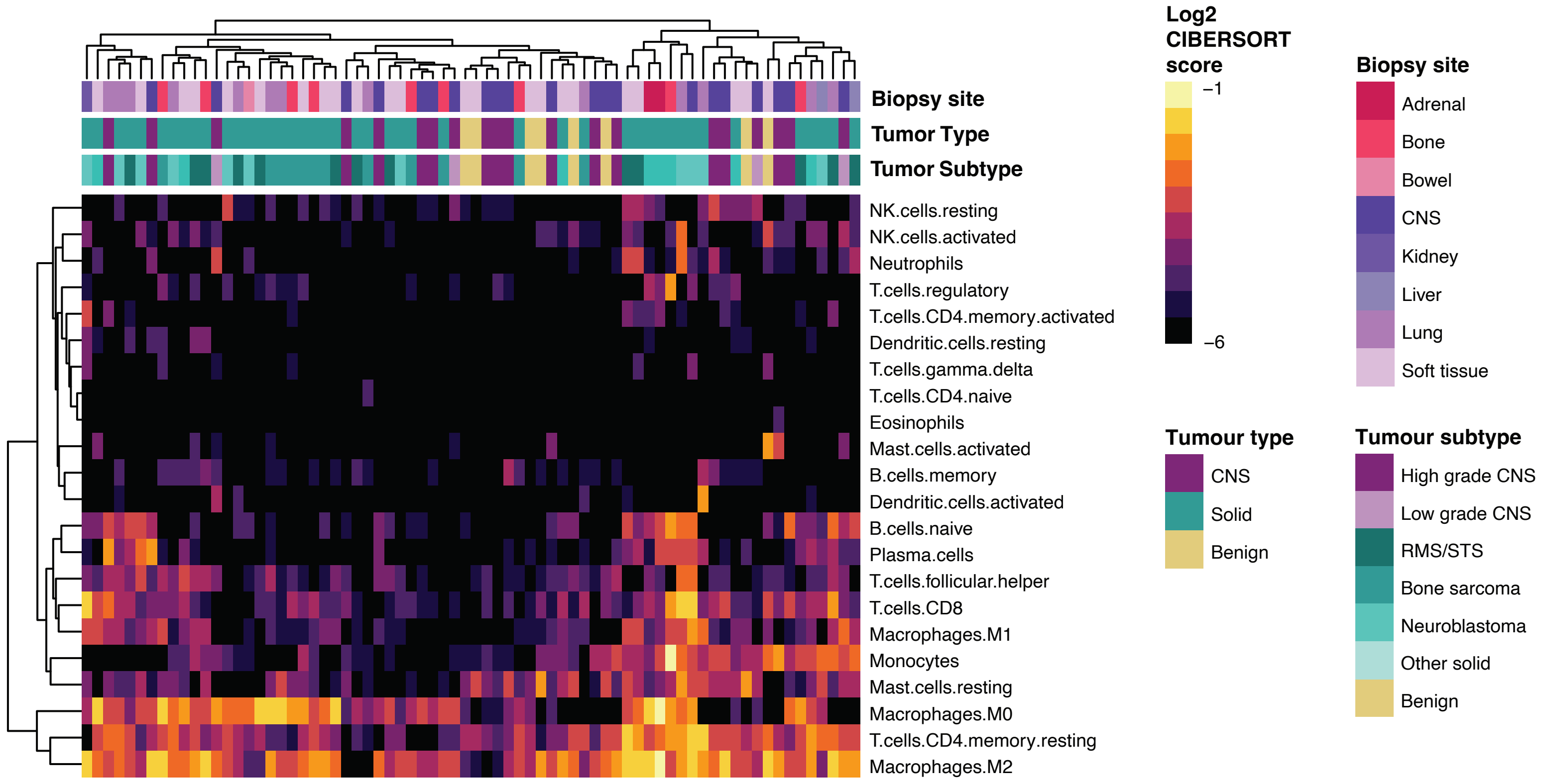

Supplementary Figure 1
Heatmap of CIBERSORT scores across 72 samples, with tracks for tumor type and sample biopsy site. Samples from lymph related biopsy sites and blood cancers are excluded.

Supplementary Figure 2

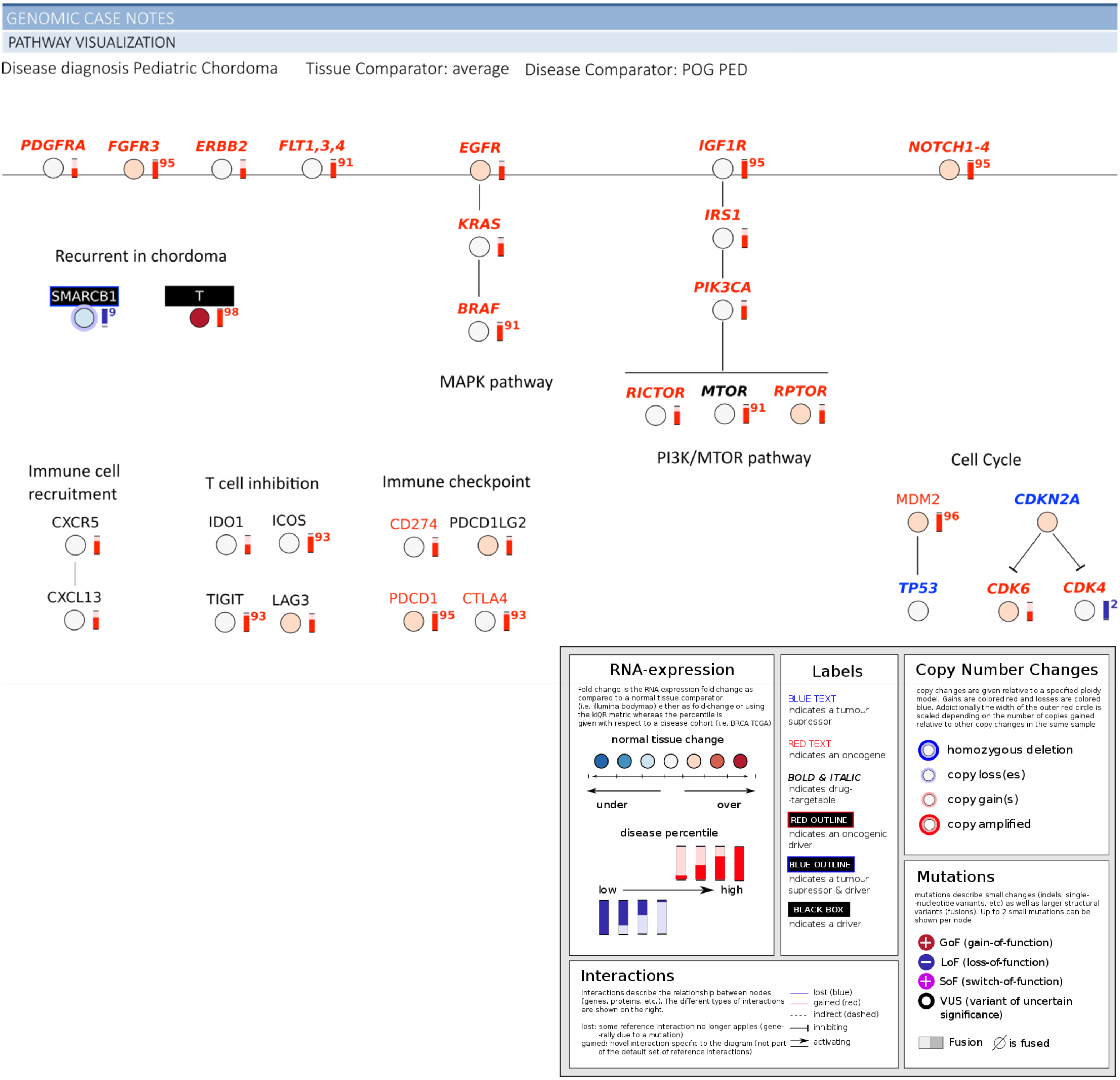

Supplementary Figure 2. The genomic pathway for a chordoma patient showing integration of DNA and RNA evidence. Normal tissue change for this sample is a comparison with Illumina body map normal tissue, and percentile calculated for disease comparison is against all pediatric POG tumors.

Supplementary Table S1. Enrolled pediatric POG participants who ultimately did not receive WGTA results (n=9).

Participant	Sample No.	Age	Time Point of Enrolment	Tumor Type	Time from Diagnosis to Biopsy (months)	No. of Biopsy Sites	Group Biopsy Site	Biopsy Type	Reason for Failure
1	N/A	16	N/A	Other solid	N/A	N/A	N/A	N/A	Not biopsied
2	1	7	Relapse/refractory	Neuroblastoma	19.8	1	Bone	Metastatic	Low tumor content
3	1	8	Relapse/refractory	Lymphoma (Hodgkin)	19.7	2	Bone marrow	Metastatic	Low DNA yield
	2				24.9		Pulmonary nodule		Low tumor content
4	1	13	Relapse/refractory	Lymphoma (Hodgkin)	8.4	1	Lymph node	Metastatic	Low DNA yield
5	1	4	Relapse/refractory	Neuroblastoma	18.8	1	Soft tissue	Metastatic	Low DNA yield
6	1	15	Relapse/refractory	Low grade CNS	9.9	1	CNS	Primary	Low tumor content
7	1	1	Relapse/refractory	Benign (Plexiform neurofibroma)	17.7	1	Soft tissue	Primary	Low tumor content
8	1	12	Relapse/refractory	Bone sarcoma	10.8	1	Pulmonary nodule	Metastatic	Low tumor content
9	1	16	Relapse/refractory	Bone sarcoma	56.6	1	Pulmonary nodule	Metastatic	Low DNA yield

Supplementary Table S2. Genes screened for germline cancer predisposition variants.
Version 1 (2013 – Oct 2016). Version 2 (Nov 2016 – Dec 2020) (– not included, • included, genes in bold were included in both versions)

gene	ver 1	ver 2
AKT1	–	•
ALK	–	•
**APC**	•	•
**ATM**	•	•
ATR	–	•
AXIN2	–	•
**BAP1**	•	•
BARD1	–	•
BLM	–	•
**BMPR1A**	•	•
**BRCA1**	•	•
**BRCA2**	•	•
BRIP1	–	•
CBL	–	•
**CDC73**	•	•
**CDH1**	•	•
CDK4	–	•
**CDKN1B**	•	•
**CDKN2A**	•	•
**CHEK2**	•	•
DICER1	–	•
**DKC1**	•	•
EGFR	–	•
**EPCAM**	•	•
ERCC2	–	•
ERCC3	–	•
ERCC4	–	•
ERCC5	–	•
ETV6	–	•
EZH2	–	•
FAM175A	–	•
FANCA	–	•
FANCC	–	•
**FH**	•	•
**FLCN**	•	•
**GATA2**	•	•
GREM1	–	•
HNF1A	–	•
HRAS	–	•
IDH1	–	•
**KIT**	•	•
**MAX**	•	•
**MEN1**	•	•
**MET**	•	•
MITF	–	•
**MLH1**	•	•
MRE11A	–	•
**MSH2**	•	•
**MSH6**	•	•
**MUTYH**	•	•
NBN	–	•
NF1	–	•
NF2	–	•
NSD1	–	•
PALB2	–	•
PAX5	–	•
**PDGFRA**	•	•
PHOX2B	–	•
PIK3CA	–	•
PMS1	–	•
**PMS2**	•	•
POLD1	–	•
POLE	–	•
**PRKAR1A**	•	•
**PTCH1**	•	•
**PTEN**	•	•
PTPN11	–	•
RAD50	–	•
RAD51	–	•
RAD51B	–	•
RAD51C	–	•
RAD51D	–	•
RB1	–	•
**RECQL4**	•	•
**RET**	•	•
RUNX1	–	•
SDHA	–	•
**SDHAF2**	•	•
**SDHB**	•	•
**SDHC**	•	•
**SDHD**	•	•
SH2D1A	–	•
**SMAD4**	•	•
SMARCA4	–	•
**SMARCB1**	•	•
**STK11**	•	•
SUFU	–	•
**TERC**	•	•
**TERT**	•	•
TGFBR1	–	•
**TINF2**	•	•
**TMEM127**	•	•
**TP53**	•	•
TSC1	–	•
TSC2	–	•
**VHL**	•	•
**WRN**	•	•
WT1	–	•

Supplementary Table S3: Number of variants with known clinical or biological relevance screened in the targeted gene report.

**Small SNVs and indels**	
Number of Genes Screened	252
Number of Variants Screened	18,239
**Fusions**	
Number of Fusion Genes Screened	447
Number of Fusion Variants Screened	825
**In house knowledgebase**	
Number of References	20,523
Number of Characterized Events	8,588

Supplementary Table S4. Level of evidence (LOE) schema.

Level of Evidence	Description
**1**	Biomarkers that predict response or resistance to therapies approved by U.S Food and Drug Administration or by Health Canada for the same tumor type (A), or a different tumor type (B)
**2**	Biomarkers that predict response or resistance to therapies based on well-powered clinical trial for the same tumor type (A), or a different tumor type (B)
**3**	Biomarkers that predict response or resistance to therapies based on small series, case-reports, or that serve as inclusion criteria for a clinical trial for the same tumor type (A), or a different tumor type (B)
**4**	Biomarkers that show plausible therapeutic significance based on preclinical studies of the same tumor type (A), or a different tumor type (B).
**5**	Novel biomarkers predicted to be oncogenic in therapeutically actionable genes, or evidence of activation of a therapeutically actionable pathway, in the same tumor type (A), or a different tumor type (B).
