# Supplementary material for: Whole genome and transcriptome integrated analyses guide clinical care of pediatric poor prognosis cancers
Source: Nat Commun. 2024 May 16;15:4165. doi: 10.1038/s41467-024-48363-5 (PMC11099106; doi:10.1038/s41467-024-48363-5)
Supplement: Supplementary file 3 — Description of Additional Supplementary Files [file 41467_2024_48363_MOESM3_ESM.pdf]

Description of Additional Supplementary files

Supplementary Data 1. All potentially therapeutically actionable genomic alterations discussed at molecular tumor boards by tumor and alteration type, gene involved, level of evidence (LOE) category, targeted therapy drug class and DNA and/or RNA evidence for all samples included in this cohort (n=83).

Supplementary Data 2. Among all participants who subsequently received molecularly informed therapy as part of clinical care (n=28), cancer type, genomic target and category (RNA/DNA), drug category, specific drug received and protocol, level of evidence, mechanism of access and best response at 6 months are described.
