# Supplementary material for: Whole genome and transcriptome integrated analyses guide clinical care of pediatric poor prognosis cancers
Source: Nat Commun. 2024 May 16;15:4165. doi: 10.1038/s41467-024-48363-5 (PMC11099106; doi:10.1038/s41467-024-48363-5)
Supplement: Supplementary file 6 — Reporting Summary [file 41467_2024_48363_MOESM6_ESM.pdf]

nature portfolio

Corresponding author(s): Deyell RJ et al

Last updated by author(s): April 12, 2024

Reporting Summary

Nature Portfolio wishes to improve the reproducibility of the work that we publish. This form provides structure for consistency and transparency in reporting. For further information on Nature Portfolio policies, see our Editorial Policies and the Editorial Policy Checklist.

Statistics

For all statistical analyses, confirm that the following items are present in the figure legend, table legend, main text, or Methods section.

n/a	Confirmed	
☐	☒	The exact sample size (*n*) for each experimental group/condition, given as a discrete number and unit of measurement
☒	☐	A statement on whether measurements were taken from distinct samples or whether the same sample was measured repeatedly
☐	☒	The statistical test(s) used AND whether they are one- or two-sided<br>*Only common tests should be described solely by name; describe more complex techniques in the Methods section.*
☐	☒	A description of all covariates tested
☒	☐	A description of any assumptions or corrections, such as tests of normality and adjustment for multiple comparisons
☐	☒	A full description of the statistical parameters including central tendency (e.g. means) or other basic estimates (e.g. regression coefficient) AND variation (e.g. standard deviation) or associated estimates of uncertainty (e.g. confidence intervals)
☐	☒	For null hypothesis testing, the test statistic (e.g. *F*, *t*, *r*) with confidence intervals, effect sizes, degrees of freedom and *P* value noted<br>*Give P values as exact values whenever suitable.*
☒	☐	For Bayesian analysis, information on the choice of priors and Markov chain Monte Carlo settings
☒	☐	For hierarchical and complex designs, identification of the appropriate level for tests and full reporting of outcomes
☒	☐	Estimates of effect sizes (e.g. Cohen's *d*, Pearson's *r*), indicating how they were calculated

Our web collection on statistics for biologists contains articles on many of the points above.

Software and code

Policy information about availability of computer code

Data collection	Sequencing data was collected internally from the Personalized Oncogenomics Program, generated by in-house Illumina sequencers as described in the Methods section. Tumor genomes were sequenced on Illumina HiSeq 2500 using v3 or v4 chemistry and paired-end 125 base reads, or on HiSeqX using v2.5 chemistry and paired-end 150 base reads. Transcriptomes were sequenced on Illumina HiSeq2500, or on NextSeq500 using v2 chemistry. Clinical data was collected from clinical health records. No external data was collected.

Data analysis	Sequence reads from normal and tumor whole genome libraries were aligned to the human reference genome (hg19) using the Burrows-Wheeler Alignment tool (v0.5.7 for up to 125 bp reads and v0.7.6a for 150 bp reads). Regions of somatic CNV and losses of heterozygosity were identified using the Hidden Markov model-based approaches CNAseq (v0.0.6) and APOLLOH (v0.1.1) respectively. Tumor purity and ploidy were identified using in-house scripts followed by manual review. Somatic SNVs were identified using two approaches: (1) putative somatic variant calls from SAMtools (v0.1.17) with subsequent scoring by machine-learning based MutationSeq (v1.0.2 and v4.3.5)41, and (2) identification and scoring with the joint caller Strelka (v1.0.6). Small indels were identified using Strelka with QSI ≥ 15. Total genomic TMB was the total number of SNVs and indels per sample and both were calculated for the whole genome. Variants were annotated to genes using SNPEff (v3.2) with the Ensembl database (v69). SVs in DNA and RNA sequence data were identified using the assembly-based tools ABySS v1.3.4 and TransABySS (v1.4.10). Putative SV calls identified from the DNA and RNA sequences were annotated against constitutional DNA to provide somatic and germline structural variant calls.RNA sequencing (RNA-Seq) reads were analyzed with JAGuaR to include alignments to a database of exon junction sequences and subsequent repositioning onto the genomic reference hg19. RNA expression was quantified using in-house scripts as reads per kilobase per million mapped reads (RPKM).<br><br>For germline DNA, SNVs and indels were called using SAMtools51, and copy number and structural variants were called using Control-FREEC52 and DELLY53, Manta54, ABySS44, and MAVIS55, respectively. SNVs and indels were annotated using SNPEff56, and region-based filtering was performed.

For manuscripts utilizing custom algorithms or software that are central to the research but not yet described in published literature, software must be made available to editors and reviewers. We strongly encourage code deposition in a community repository (e.g. GitHub). See the Nature Portfolio guidelines for submitting code & software for further information.

Data

Policy information about availability of data

All manuscripts must include a data availability statement. This statement should provide the following information, where applicable:

- Accession codes, unique identifiers, or web links for publicly available datasets
- A description of any restrictions on data availability
- For clinical datasets or third party data, please ensure that the statement adheres to our policy

The tumor WGS and RNA-Seq raw data generated in this study have been deposited in the European Genome-phenome Archive [https://ega-archive.org/studies/EGAS00001006967]. Three other patients have been previously deposited and can be accessed at https://ega-archive.org/datasets/EGAD00001008012, https://ega-archive.org/datasets/EGAD00001008013 and https://ega-archive.org/datasets/EGAD00001004712. The WGS and RNA-Seq data are available under controlled access to ensure strict confidentiality. Access can be obtained by submitting a request to our Data Access Committee [https://ega-archive.org/dacs/EGAC00000000011].

Publicly available transcriptome sequencing data from normal and tumor tissues that are used for gene expression analysis is available at, Illumina BodyMap 2.0 (https://www.ensembl.info/2011/05/24/human-bodymap-2-0-data-from-illumina/), the Genotype-Tissue Expression (GTEx) Project (https://gtexportal.org/home/), The Cancer Genome Atlas (TCGA,https://portal.gdc.cancer.gov/), Treehouse Childhood Cancer Initiative (https://treehousegenomics.soe.ucsc.edu/public-data/), and the TARGET program (https://www.cancer.gov/ccg/research/genome-sequencing/target). Pediatric sequencing data was compared to our adult pan-cancer cohort on POG, for which data has also been deposited in the European Genome-phenome Archive (Accession # EGAS00001001159, https://web2.ega-archive.org/studies/EGAS00001001159) .

Research involving human participants, their data, or biological material

Policy information about studies with human participants or human data. See also policy information about sex, gender (identity/presentation), and sexual orientation and race, ethnicity and racism.

Reporting on sex and gender	Biologic sex was collected from patient health records with patient / parent consent as part of baseline demographic characteristics and eligibility was not restricted by sex or gender. Sex is only reported in aggregate as part of descriptive characteristics for the cohort. (Table 1)
Reporting on race, ethnicity, or other socially relevant groupings	Race, ethnicity or other socially relevant groupings were not collected as part of clinical data for this cohort.
Population characteristics	In the final study cohort (n=79), 43% of the participants were female and the median age at cancer diagnosis and study enrollment was 8.8y (range 0 to 20.7y) and 13.4y (range 0.5 to 21.2y), respectively. The cohort included 46 (58%) solid, non-CNS, 19 (24%) CNS, 8 (10%) benign, and 6 (8%) hematologic malignancies (Table 1). The most common tumor types were soft tissue and bone sarcoma (n=24), high grade primary CNS tumors (n=16), and neuroblastoma (n=9), with samples obtained from a range of biopsy sites.
Recruitment	Eligible patients were identified by their treating oncologist at the only pediatric quaternary health care center in British Columbia. It is possible that not all eligible patients were approached due to selection bias of the primary oncologist. However, this risk is minimal as our program systematically reviews and identifies all hard to treat pediatric cancer patients in a group setting. If some patients were not approached, it may result in our final cohort not being fully representative of our poor prognosis pediatric cancer population. If patients / parents were deemed likely eligible and were interested in learning more about the study, a consent and assent meeting was set up with the study team. Following the consent process, participants were enrolled if eligibility was confirmed.
Ethics oversight	The study was approved by the BC Children's and Women's Research Ethics Board (#H13-01640).

Note that full information on the approval of the study protocol must also be provided in the manuscript.

Field-specific reporting

Please select the one below that is the best fit for your research. If you are not sure, read the appropriate sections before making your selection.

☒ Life sciences ☐ Behavioural & social sciences ☐ Ecological, evolutionary & environmental sciences

For a reference copy of the document with all sections, see nature.com/documents/nr-reporting-summary-flat.pdf

Life sciences study design

All studies must disclose on these points even when the disclosure is negative.

Sample size	The study describes a convenience sample based on inclusion of all enrolled participants for descriptive primary outcomes.
Data exclusions	No data was excluded.
Replication	Data was not replicated or validated in this study. Replication of whole genome and transcriptome sequencing was cost prohibitive.
Randomization	There was no randomization involved in this non-interventional study.
Blinding	This non-interventional study did not include blinding.

Reporting for specific materials, systems and methods

We require information from authors about some types of materials, experimental systems and methods used in many studies. Here, indicate whether each material, system or method listed is relevant to your study. If you are not sure if a list item applies to your research, read the appropriate section before selecting a response.

Materials & experimental systems

n/a	Involved in the study	
☒	☐	Antibodies
☒	☐	Eukaryotic cell lines
☒	☐	Palaeontology and archaeology
☒	☐	Animals and other organisms
☐	☒	Clinical data
☒	☐	Dual use research of concern
☒	☐	Plants

Methods

n/a	Involved in the study	
☒	☐	ChIP-seq
☒	☐	Flow cytometry
☒	☐	MRI-based neuroimaging

Clinical data

Policy information about clinical studies

All manuscripts should comply with the ICMJE guidelines for publication of clinical research and a completed CONSORT checklist must be included with all submissions.

Clinical trial registration	This was not an interventional clinical trial and so it was not regsitered separately.
Study protocol	Full study protocol available is REB approved and available on request.
Data collection	Patients with relapsed, refractory or hard to treat cancer were enrolled between September 2013 and July 2019. All were patients at the only quaternary pediatric oncology program in British Columbia.
Outcomes	Outcomes were primarily descriptive with respect to actionable germline and somatic findings. Data was also collected among patients who subsequently pursued molecularly targeted therapy and response and survival outcomes are described, by level of evidence. Five patients accessed subsequent therapy via enrollment on clinical trials which have all been previously published (NCT02343718, NCT02303028, NCT02637687 and NCT03416530).
